# INNOVATIONS IN TECHNOLOGY FOR SOCIAL ENGAGEMENT AMONG OLDER ADULTS AND TO IMPROVE CAREGIVER COMMUNICATION AND TRAINING

**DOI:** 10.1093/geroni/igac059.927

**Published:** 2022-12-20

**Authors:** Hongdao Meng, Sean Barbeau, Mingyang Li, Cassidy Doyle, Kelly Wiechart, Robert Hammond, William Kearns

**Affiliations:** University of South Florida, Tampa, Florida, United States; University of South Florida, Tampa, Florida, United States; University of South Florida, Tampa, Florida, United States; University of South Florida, Tampa, Florida, United States; KellyW Consulting, Tampa, Florida, United States; University of South Florida, Tampa, Florida, United States; University of South Florida, Tampa, Florida, United States

## Abstract

Music-based interventions (MBIs) have been shown to benefit persons with dementia. To develop new MBIs and build the evidence base for motor, cognitive, emotional, and behavioral outcomes, accurate measures of music exposure (e.g., content, dose, and duration) are critically important. However, most commercial music services require internet access and/or subscription fees without providing convenient ways for researchers to access music exposure data. Our research team developed the open-source MUsic to Support Engagement and Resilience (MUSER) Android application with an associated data processing engine. Caregivers can use MUSER to deliver individualized music selections via smartphones/tablets, with music exposure data being generated for research use. We used the Kotlin programming language for application development and Java/Python/Microsoft BI for data processing and analytics instrumentation. The MUSER application was tested for music exposure measurement feasibility with 16 users (ten research team members and six family caregivers of persons with dementia) for two weeks between July and November of 2021. Semi-structured interviews were conducted pre-post application testing to gather qualitative and quantitative data to enrich the understanding of user experiences. Family caregivers had a mean age of 57.8 (SD=26.2; range 20-77). The average number of listening days was 6 (SD=2.5), and the average music listening duration was 203 minutes (SD=123.2). Caregivers played an average of 52 (SD=36.9) songs from 10 (SD=4.2) albums involving 34 (SD=36.3) artists. Given the ease of deployment and integrated data collection and reporting capabilities, this application supports collaborative software development among researchers to advance MBI research.

